# Effect of Cyclic Shear Fatigue under Magnetic Field on Natural Rubber Composite as Anisotropic Magnetorheological Elastomers

**DOI:** 10.3390/polym14091927

**Published:** 2022-05-09

**Authors:** Jeong-Hwan Yoon, Seung-Won Lee, Seok-Hu Bae, Nam-Il Kim, Ju-Ho Yun, Jae-Hum Jung, Young-Gil Kim

**Affiliations:** 1 Energy Materials R&D Centre, Materials Technology R&D Division, Korea Automotive Technology Institute, 303 Pungse-ro, Pungse-myeon, Dongnam-gu, Cheonan-si 31214, Chungnam, Korea; swlee1@katech.re.kr (S.-W.L.); shbae@katech.re.kr (S.-H.B.); nikim@katech.re.kr (N.-I.K.); jhyun@katech.re.kr (J.-H.Y.); 2 Material Development Team, Deaheung Rubber & Technology Co., Ltd., 436 Seobu-ro, Jillye-myeon, Gimhae-si 50872, Gyeongnam, Korea; jaehum.jung@dhrnt.com (J.-H.J.); younggil.kim@dhrnt.com (Y.-G.K.)

**Keywords:** natural rubber, carbonyl iron particle, anisotropic, magnetorheology, shear fatigue

## Abstract

With the development and wide applicability of rubber materials, it is imperative to determine their performance under various conditions. In this study, the effect of cyclic shear fatigue on natural-rubber-based anisotropic magnetorheological elastomer (MRE) with carbonyl iron particles (CIPs) was investigated under a magnetic field. An anisotropic MRE sample was prepared by moulding under a magnetic field. Cyclic shear fatigue tests were performed using a modified electromechanical fatigue system with an electromagnet. The storage modulus (G′) and loss factor in the absence or presence of a magnetic field were measured using a modified dynamic mechanical analysis system. Under a magnetic field, fatigue exhibited considerable effects to the MRE, such as migration and loss of magnetised CIPs and suppressed increase in stiffness by reducing the energy loss in the strain cycle. Therefore, the G′ of the MRE after fatigue under a magnetic field was lower than that after fatigue in the zero field. The performance of the MRE, such as absolute and relative magnetorheological effects, decreased after subjecting to cyclic shear fatigue. In addition, all measured results exhibited strain-dependent behaviour owing to the Payne effect.

## 1. Introduction

Rubbery materials with viscoelastic properties exhibit complex behaviour owing to their deformability, stress softening, and time-dependent attributes; thus, they are widely used in various industrial applications such as seals, dampers, bushings, and tires, among others [1,2,3,4,5]. As rubber products are exposed to different environmental conditions and subjected to cyclic loading, they often fail owing to nucleation and the presence of defects [6,7]. In addition, the stress–strain curve of a rubber material subjected to cyclic loading exhibits a hysteresis loop, indicating energy loss owing to its viscoelasticity. Most of this energy loss is eventually dissipated into heat. When the heat generated by cyclic loading does not exit the material and accumulates, the temperature of the material increases, resulting in fatigue failure or changes in the material properties [8,9,10,11,12,13].

The unique rheological properties of magnetorheological elastomers (MREs) can be easily tuned when exposed to an external magnetic field, making them suitable for advanced rubber products, such as adaptive dampers and stiffness-tuneable mounts. Natural rubber (NR) exhibiting high mechanical properties can contain high content of magnetic particle to improve the MRE’s performances, while soft carbonyl iron particles (CIPs) are widely used because of their high permeability, high magnetic saturation, and low remnant magnetization. On the other hand, MRE containing a large amount of filler has a relatively low fatigue resistance. There have been ample studies on various approaches to improve the performance of MREs. Recently, researchers have reported the fatigue behaviour of MREs under a magnetic field [14,15,16,17,18,19,20,21]. Zhou et al. [22] reported that the fatigue life of an MRE increases when fatigue is induced under an external magnetic field. Lian et al. [23] demonstrated the effect of repetition of a magnetic field on the fatigue behaviour, hysteresis loss, and storage modulus of MREs. Meanwhile, most studies on the durability of MRE have focused on monitoring the change of tensile strength and compressive load [24,25,26]. However, the effect of an external magnetic field on the property changes of MRE during cyclic fatigue needs to be further investigated for the actual application as engine mount.

In this study, an anisotropic MRE was fabricated using NR, which is widely used as the matrix for MRE, and CIPs. Cyclic shear fatigue and strain amplitude sweep tests were performed to investigate the effect of the magnetic field on the prepared samples.

## 2. Materials and Methods

NR (CV-60, Standard Vietnam Rubber) was used as the rubber matrix. Carbon black (N990, Cancarb, Alberta, Canada) was used as the reinforcement to improve the mechanical properties. Spherical CIPs (CC grade, BASF, Ludwigshafen, Germany) with a mean particle size of 3–5 μm were used as the soft magnetic particles. Processing oil (N-2, Michang, Busan, Korea), sulphur (MIDAS SP 325, Miwon, Anyang, Korea), thiuram disulphide (ORICELL TT, OCI, Seoul, Korea), and sulphonamide (ORICELL CZ, OCI, Seoul, Korea) were used as the curing agents and accelerators.

A Banbury mixer (HYB-3L, Hyupyoung Machinery, Kimpo, Korea) was used to prepare the MRE samples. First, 150 parts per hundred rubber (phr) CIPs, 15 phr carbon black, 20 phr processing oil, 1.5 phr sulphur, 1.5 phr thiuram disulphide, and 2.0 phr sulphonamide were compounded with NR at 50 °C for 15 min. The resulting mixture was moulded into disks with a thickness of 3.0 mm and diameter of 7.0 mm under approximately 10 MPa in a magnetic field with an intensity of 1000 mT at 160 °C for 400 s. As a result, an anisotropic MRE sample with CIPs aligned out-of-plane along the magnetic field direction in the matrix was obtained.

**Figure 1:**
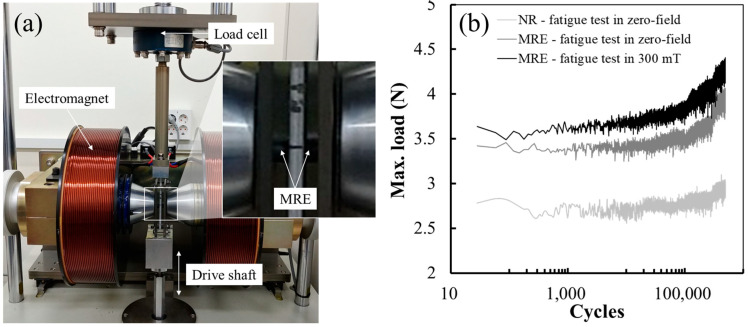
(**a**) Photos of the modified electromechanical fatigue system equipped with an electromagnet. (**b**) Maximum recorded load during cyclic shear fatigue of the NR and anisotropic MRE samples.

Cyclic shear fatigue tests were performed using a modified electromechanical fatigue system (Fatigue tester, Daekyung engineering, Bucheon, Korea) equipped with an electromagnet that can generate a magnetic field under uniaxial shear loading (Figure 1a). The shear direction was perpendicular to the direction of the magnetic field and aligned CIPs. The system was designed to maintain a constant magnetic flux density during fatigue tests. A relatively weak magnetic flux density of 300 mT was applied to confirm the effect of the magnetic field on the MRE and generate a constant magnetic field for up to 500,000 fatigue cycles. Based on the test conditions, such as engine mounts where MRE can be applied, a strain amplitude of 50% and frequency of 5 Hz at an ambient temperature of 23 °C was set. The maximum load applied to the sample was recorded. The changes in the morphology, specific gravity, and dynamic viscoelastic properties of the anisotropic MRE samples were measured after subjecting them to cyclic shear fatigue. The cross-sectional morphologies of the MREs were observed using a laser confocal microscope (OLS5000, Olympus, Shinjuku, Japan). An analytical balance (MS204TS, Mettler Toledo, Greifensee, Switzerland) was used to obtain the specific gravity according to ISO 1183-1. The rheological properties of the samples were examined using the modified dynamic mechanical analysis system presented in our previous work [27]. Three types of properties were measured: storage modulus, loss modulus, and loss factor. When an external magnetic field was applied, the direction of the magnetic field was perpendicular to the surface of the tested sample and parallel to the aligned structure of the CIPs within the anisotropic MRE. Thus, the modulus measured under the magnetic field detected the response of the sample in the perpendicular direction. The strain amplitude sweep test was performed by varying the strain from 0.1 to 5.0% with 1 Hz at an ambient temperature of 23 °C. In the on-state measurement, a magnetic flux density of 1200 mT was applied for the sufficient saturated magnetisation of the magnetic particles.

## 3. Results and Discussion

### 3.1. Cyclic Shear Fatigue Test

Figure 1b shows the maximum recorded load with the increasing number of cycles for the NR and MRE samples. The NR sample had the same composition as that of the MRE sample, except for the presence of CIPs in the latter. The MRE sample was subjected to zero and external magnetic fields. The load of the samples increased with the increase in the number of cycles. In particular, the load of the MRE sample was higher than that of the NR sample. Owing to the higher stiffness of the MRE sample, a larger maximum load is required for its deformation. Moreover, the maximum load increases further under an external magnetic field, consistent with the known effect of an external magnetic field on the MRE sample [1,27,28,29,30]. Meanwhile, the material exhibited variable response to the fatigue cycles at a fixed strain. The load deviations increased as the number of fatigue cycles increased. This phenomenon is associated to the stress softening due to the sliding between the matrix chains and filler particles, hysteretic heating of the rubber materials by the energy loss in the strain cycle, and accumulation of crystallisation under non-relaxing conditions. Stress softening under cyclic loading, especially for filled rubber composites, has been described by the Mullins and Payne effects [8,9,10,11,12,13,31,32,33,34,35,36,37].

Softening and hardening are complex events that occur in samples during the cyclic shear fatigue test, and these changes may affect the rheological properties of the MRE. Interestingly, the CIPs embedded in the MRE were attracted to the magnetic field and were released during the fatigue test under an external magnetic field. The detachment of the CIPs weakens physical bonds between the NR matrix and CIPs owing to the relative movement of magnetised CIPs and the large strain amplitude applied to the MRE [18,38,39,40]. This can explain the slightly lower increase in the maximum recorded load under a magnetic field that in a zero field as the number of fatigue cycles increased.

### 3.2. Morphology

**Figure 2:**
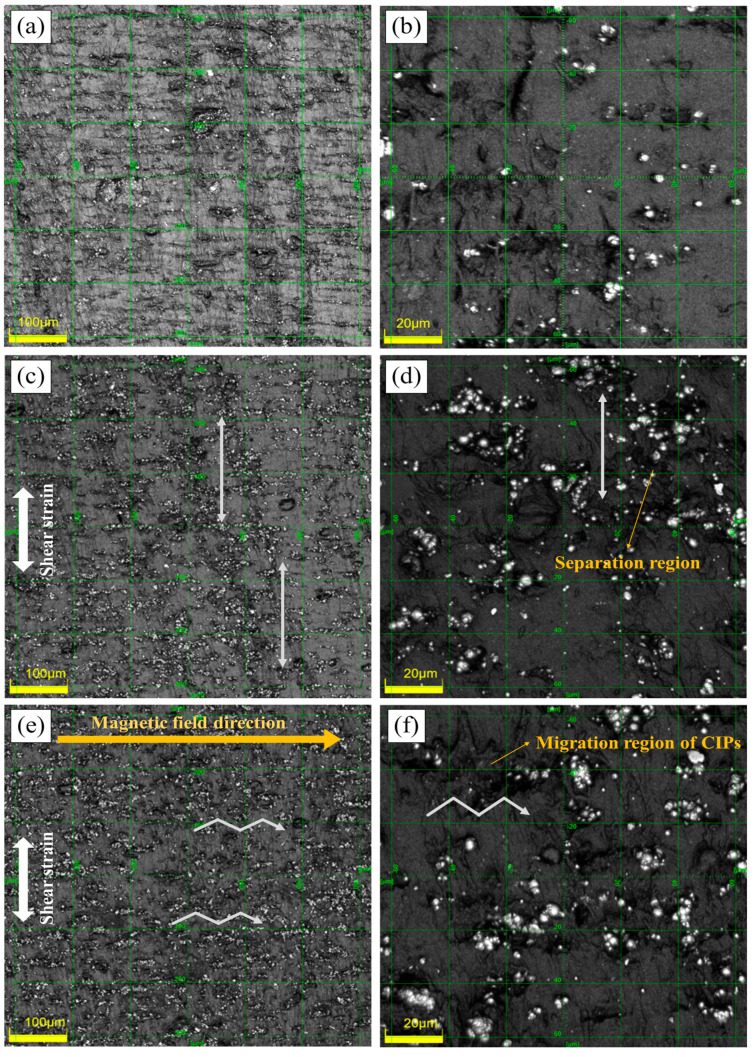
Microscope images with (**a**) 20× and (**b**) 100× magnification of the initial MRE cross-section; and (**c**) 20× and (**d**) 100× magnification of the MRE cross-section after the cyclic shear fatigue in the zero field; and (**e**) 20× and (**f**) 100× magnification of the MRE cross-section after 500,000 shear fatigue cycles under magnetic field. The bright parts represent the magnetic particles embedded in the NR. The white arrows represent the direction of separation or migration regions.

The laser confocal microscope images of the cross-sectional morphologies of the initial MRE samples and after the fatigue tests are shown in Figure 2. Initially, the CIPs were aligned and embedded in NR matrix with some of the agglomerations in the magnetic field direction. However, in the samples subjected to fatigue, most of the particles were separated from the surrounding NR matrix, as can be seen in the dark area around the particles in Figure 2c–f. Different morphologies were observed based on the presence or absence of a magnetic field. Figure 2c shows that the separation regions are mainly arranged in the shear strain direction. Meanwhile, most of the CIPs are dissociated from the surrounding NR matrix in this direction. However, when shear fatigue was applied under a magnetic field, separation occurred in the magnetic field direction, as shown in Figure 2e,f. Although the CIPs are separated from the surrounding NR matrix by the shear strain, interparticle forces between the magnetised CIPs maintained the physical bond with the NR matrix. However, the shear strain continuously separated the magnetised CIPs and NR matrix, along with the interparticle forces generated the migration regions by shifting the CIPs, as shown in Figure 2f. Therefore, the separation regions were observed in the shear strain and magnetic field directions as in zig-zag arrows. Some CIPs exhibited agglomerated morphologies in the direction of the interparticle forces. In addition, the magnetised CIPs on the surface of the MRE can be attracted to and lost to an external electromagnet when separated from the NR matrix.

### 3.3. Specific Gravity

Table 1 lists the specific gravities of the MRE samples before and after subjecting them to cyclic shear fatigue. The specific gravity of the MRE sample prior to fatigue testing was 2.586 g/cm^3^. After subjecting the MRE sample to fatigue for up to 500,000 cycles in the zero field, the specific gravity of the sample remained constant. Meanwhile, the specific gravity of the MRE sample subjected to fatigue under a magnetic field decreased with the increase in the fatigue cycles. This indicates changes in the MRE composition. In particular, the change in specific gravity of the MRE, as confirmed during the cyclic shear fatigue test, can be ascribed to the loss of the CIPs with a specific gravity of 7.86 g/cm^3^.

### 3.4. Rheological Properties

**Figure 3:**
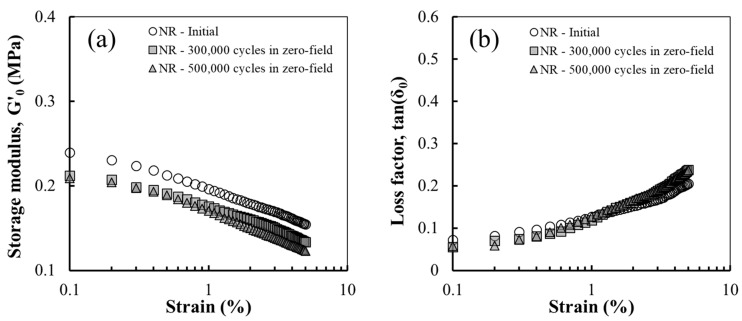
(**a**) G′_0_ and (**b**) tan(δ_0_) of the NR before and after cyclic shear fatigue.

Strain amplitude sweep tests were performed to investigate the change in the dynamic viscoelastic modulus of the MREs under cyclic shear fatigue. Figure 3 shows the storage modulus (G′_0_) and loss factor (tan(δ_0_)) of the NR sample as a function of the strain amplitude in the zero-field after 0, 300,000, and 500,000 fatigue cycles. As shown in Figure 3a, the G′_0_ of the NR sample decreased with increasing strain amplitude owing to the Payne effect and decreased with fatigue. In addition, tan(δ_0_) increased as the number of fatigue cycles increased, as shown in Figure 3b. These results are consistent with the generally known behaviour, that is, the remarkable decrease in the storage modulus. As the number of cycles increased, damage gradually propagated, thereby decreasing G′_0_ and increasing tan(δ_0_) because the friction inside the material causes more energy dissipation [41,42].

**Figure 4:**
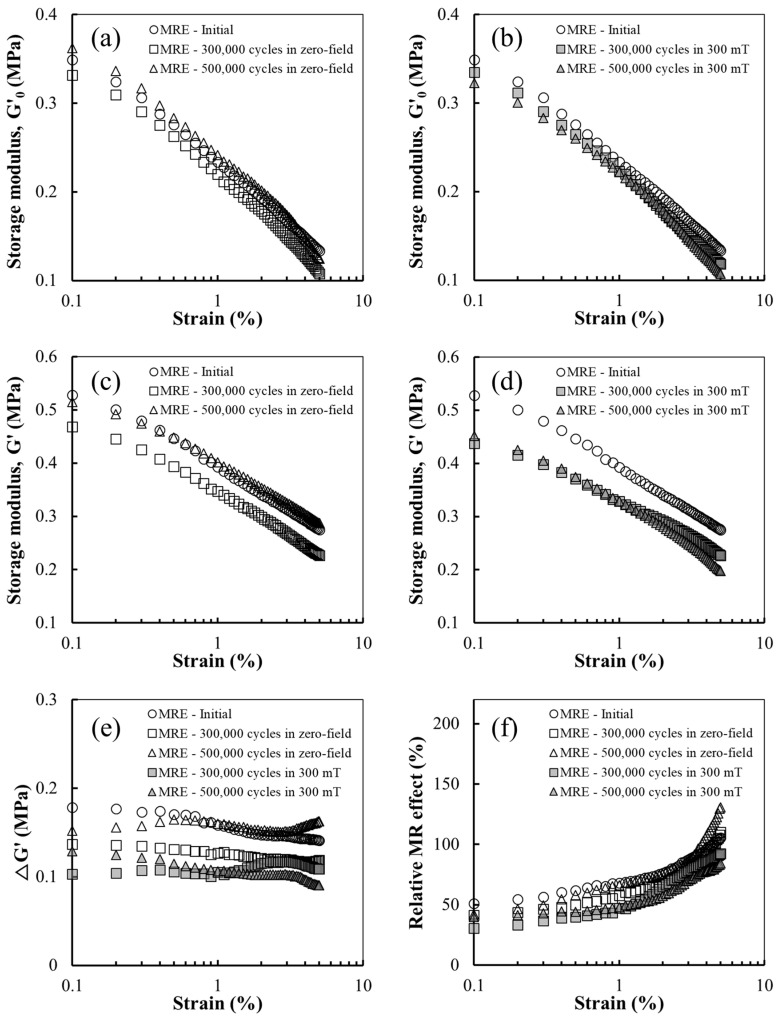
Off-state G′_0_ of the MRE sample before and after being subjected to cyclic shear fatigue in the (**a**) absence and (**b**) presence of a magnetic field. On-state G′ of the MRE sample before and after cyclic shear fatigue in the (**c**) absence and (**d**) presence of magnetic field. (**e**) Absolute and (**f**) relative MR effects of the MRE samples.

Figure 4 shows the rheological properties of the MRE sample in the absence (off-state) and presence (on-state) of the magnetic field under cyclic shear fatigue. After 300,000 cycles, the G′_0_ of the MRE sample was lower than that of the initial sample, exhibiting a behaviour similar to the NR sample. After 500,000 cycles, G′_0_ increased again. This trend can be attributed to the increase in the maximum load during the cyclic shear fatigue test. The MRE sample has more pronounced hysteretic heating and crystallisation accumulation, resulting in its higher stiffness. In contrast, the G′_0_ value of the MRE sample before and after being subjected to fatigue under a magnetic field exhibits a slightly different trend, as shown in Figure 4b. After 500,000 cycles under a magnetic field, the G′_0_ of the sample decreased more than that of the sample after 300,000 cycles. This trend can be ascribed to the relative movement of the CIPs limited by the magnetic field and loss of CIPs. In particular, the limited relative movement of the CIPs can suppress the increase in stiffness of the MRE by reducing the energy loss in the strain cycle. In addition, this decreases the softness of the MRE because of the CIPs lost during the cyclic shear fatigue test.

The on-state G′ trend of the MRE before and after being subjected to cyclic shear, as shown in Figure 4c,d, is similar to that of the off-state G′_0_ in Figure 4a,b, respectively. However, the difference of the G′_0_ values before and after being subjected to fatigue is larger. This suggests the effect of fatigue on the modulus stored in the restrained matrix generated by the interparticle forces. Therefore, the magnetorheological (MR) performance of an MRE can be altered by cyclic shear fatigue as it is dependent on the capacity of the modulus stored in the restrained matrix [43]. When cyclic shear fatigue is applied under a magnetic field, the decrease in G′ is more pronounced owing to agglomeration caused by the migration of the CIPs, as shown in Figure 2f. This is ascribed to the increased distance between the CIP aggregates as the CIPs move, which can weaken the interparticle forces or reduce the area of the restrained matrix around the magnetised CIPs.

The performance of the MRE sample is usually evaluated by the absolute and relative MR effects, which represent the change in the storage modulus under a magnetic field [44,45,46,47]. The absolute MR effect (ΔG′) represents the difference between G′ and G′_0_. The relative MR effect is the percentage of ΔG′ and G′_0_, as:

(1) $$\mathrm{Relative}\;\mathrm{MR}\;\mathrm{effect}\;=\frac{\Delta G\text{’}}{{G\text{’}}_{0}}\times 100\%.$$

Figure 4e,f shows the absolute and relative MR effects of the MRE sample before and after subjecting it to fatigue, respectively. Except for the sample after 500,000 cycles in the zero field, which has the highest G′, the ΔG′ values of all samples after fatigue were lower than that of the initial sample. Similar to the results of the G′ values, lower ΔG′ values were obtained for the samples after fatigue. As G′_0_ decreased with increasing strain amplitude owing to the Payne effect, the relative MR effect was strain-dependent, that is, it increased with increasing strain amplitude.

**Figure 5:**
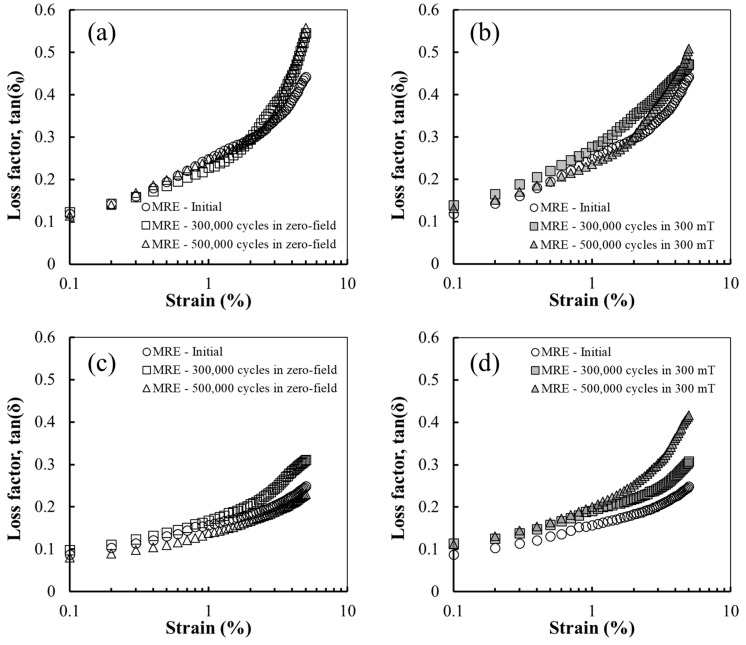
Off-state tan(δ_0_) of the MRE sample before and after cyclic shear fatigue in the (**a**) absence and (**b**) presence of a magnetic field. On-state tan(δ) of the MRE sample before and after cyclic shear fatigue in the (**c**) absence and (**d**) presence of magnetic field.

The tan(δ_0_) of the MRE increased with increasing energy dissipation after subjecting it to cyclic shear fatigue, as shown in Figure 5a,b, which is consistent with the results of the NR sample in Figure 3. As shown in Figure 5a, the tan(δ_0_) value of the sample after fatigue in the zero field at a lower strain amplitude was slightly lower than that of the initial sample. In contrast, the tan(δ_0_) value of the sample at a lower strain amplitude after 300,000 cycles under a magnetic field was higher than that of the initial sample, as shown in Figure 5b, which can be ascribed to the higher energy dissipation owing to the migration of magnetised CIPs. The tan(δ_0_) behaviour with the strain amplitude of the sample after 500,000 cycles under a magnetic field is expected to vary under the influence of further migration and CIP losses. Figure 5c,d shows the tan(δ) value measured in the on-state condition, which exhibits a different behaviour from that of tan(δ_0_). The loss factor based on the relative movement between the CIPs and matrix in the on-state was lower than that in the off-state because the interparticle forces between the CIPs inside the rubber matrix limit the relative movement of the CIPs, thereby reducing the energy dissipation [18,38,39,40]. Therefore, the tan(δ) values of all samples were lower than of tan(δ_0_). Moreover, the tan(δ) of all samples, except for that after 500,000 cycles in the zero field, was higher than that of the initial sample. The lower tan(δ) of the sample after 500,000 cycles in the zero field is attributed to the reduced sliding between the matrix chains and particles due to the expansion of the separation region and limited relative movement of the magnetised CIPs [31]. The highest tan(δ) was obtained after 500,000 cycles under a magnetic field, as shown in Figure 5d. This can be explained by the agglomerate formation and particle loss due to the migration of the CIPs after cyclic shear fatigue owing to the low G′. The internal changes in the MRE after fatigue increased the distance between the CIPs, thereby weakening the interparticle forces. This reduces the force limiting the relative movement of the magnetised CIPs, thereby increasing energy dissipation. In addition, the cyclic shear fatigue under a magnetic field reduced the area of the separation region between the particles and matrix, and some CIPs maintained the physical bonds with the surrounding matrix even after fatigue, as shown in Figure 2f. Consequently, tan(δ) tends to increase as the number of fatigue cycles increases under a magnetic field.

## 4. Conclusions

In this study, cyclic shear fatigue and strain amplitude sweep tests were performed on NR-based MRE samples to clarify the changes in the dynamic viscoelastic properties under fatigue. When the cyclic shear fatigue tests were performed in the zero field and an external magnetic field, the material response to the fatigue cycles at a fixed strain was not constant. In addition, the maximum recorded load increased with the increase in the number of fatigue cycles. Under a magnetic field, the CIPs on the MRE surface were extracted with reduced specific gravity from 2.586 to 2.565 g/cm^3^, where the migration of the CIPs was observed in the cross-sectional morphologies. The absence or presence of a magnetic field during fatigue affected the changes in the G′_0_ of the MRE. In addition, the magnetic field suppressed the increase in stiffness of the MRE by reducing the energy loss in the strain cycle. However, the magnetic field induced the migration and loss of CIPs, and consequently, decreased the absolute and relative MR effects of the MRE after fatigue under a magnetic field. As the relative movement of the CIPs in the MRE is restricted by the magnetic field, tan(δ) is lower than tan(δ_0_). However, both tan(δ_0_) and tan(δ) increased after fatigue. After 500,000 fatigue cycles in the zero field, the sliding between the matrix chains and particles decreased owing to the expansion of the separation region, resulting in a lower tan(δ) than the initial sample. After fatigue test, the maximum G′ decreased slightly from 0.53 MPa to 0.51 MPa and 0.45 MPa over the whole strain amplitude range investigated in absence or presence of a magnetic field, respectively. No significant change was also observed in the maximum tan(δ) value. This indicates the effect of the cyclic shear fatigue accumulated in the absence or presence of a magnetic field on the magnetorheological properties of the MRE sample. This is also an important factor to be considered in the application of MRE for further research.

## Figures and Tables

**Table 1:** Specific gravity of the MRE sample before and after cyclic shear fatigue in the absence or presence of a magnetic field.

Number of Fatigue Cycles	0	Zero-Field		Magnetic Field (300 mT)	
		300,000	500,000	300,000	500,000
Specific gravity (g/cm^3^)	2.586	2.581	2.583	2.577	2.565

## Data Availability

The data presented in this study are available on request from the corresponding author.

## References

[1] Hosseini S.M., Shojaeefard M.H., Saeidi G.H. (2021). Fatigue life prediction of magneto-rheological elastomers in magnetic field. Mater. Res. Express..

[2] Laraba-Abbes F., Ienny P., Piques R. (2003). A new ‘Tailor-made’ methodology for the mechanical behaviour analysis of rubber-like materials: II. Application to the hyperelastic behaviour characterization of a carbon-black filled natural rubber vulcanizate. Polymer.

[3] Wang X.L., Liao M.Y., Xu Y., Liu X.A. (2018). Fatigue crack propagation characteristics of rubbery materials under variable amplitude loading. Results Phys..

[4] Tee Y.L., Loo M.S., Andriyana A. (2018). Recent advances on fatigue of rubber after the literature survey by Mars and Fatemi in 2002 and 2004. Int. J. Fatigue.

[5] Shangguan W.B., Wang X.L., Deng J.X., Rakheja S., Pan X.Y., Yu B. (2014). Experiment and modeling of uniaxial tension fatigue performances for filled natural rubbers. Mater. Des..

[6] Gent A.N., Lindley P.B., Thomas A.G. (1964). Cut growth and fatigue of rubbers. I. The relationship between cut growth and fatigue. J. Appl. Polym. Sci..

[7] Yeoh O.H. (2001). Analysis of deformation and fracture of ‘pure shear’ rubber testpiece. Plast. Rubber. Compos..

[8] Luo W., Huang Y., Yin B., Jiang X., Hu X. (2020). Fatigue life assessment of filled rubber by hysteresis induced self-heating temperature. Polymers.

[9] Le S.V., Marco Y., Calloch S., Doudard C., Charrier P. (2010). Fast evaluation of the fatigue lifetime of rubber-like materials based on a heat build-up protocol and micro-tomography measurements. Int. J. Fatigue.

[10] Luo W., Yin B., Hu X., Zhou Z., Deng Y., Song K. (2018). Modeling of the heat build-up of carbon black filled rubber. Polym. Test..

[11] Zhi J., Wang S., Zhang M., Wang H., Lu H., Lin W. (2019). Numerical analysis of the dependence of rubber hysteresis loss and heat generation on temperature and frequency. Mech. Time-Depend. Mat..

[12] Le S.V., Marco Y., Calloch S., Charrier P., Taveau D. (2013). Heat build-up of rubber under cyclic loadings: Validation of an efficient demarch to predict the temperature fields. Rubber Chem. Technol..

[13] Katunin A. (2019). Criticality of the self-heating effect in polymers and polymer matrix composites during fatigue, and their application in non-destructive testing. Polymers.

[14] Schümann M., Odenbach S. (2017). In-situ observation of the particle microstructure of magnetorheological elastomers in presence of mechanical strain and magnetic fields. J. Magn. Magn. Mater..

[15] Perales-Martínez I.A., Palacios-Pineda L.M., Lozano-Sánchez L.M., Martínez-Romero O., Puente-Cordova J.G., Elías-Zúñiga A. (2017). Enhancement of a magnetorheological PDMS elastomer with carbonyl iron particles. Polym. Test..

[16] Burgaz E., Goksuzoglu M. (2020). Effects of magnetic particles and carbon black on structure and properties of magnetorheological elastomers. Polym. Test..

[17] Cvek M., Kracalik M., Sedlacik M., Mrlik M., Sedlarik V. (2019). Reprocessing of injection-molded magnetorheological elastomers based on TPE matrix. Compos. B. Eng..

[18] Yu M., Qi S., Fu J., Zhu M., Chen D. (2017). Understanding the reinforcing behaviors of polyaniline-modified carbonyl iron particles in magnetorheological elastomer based on polyurethane/epoxy resin IPNs matrix. Compos. Sci. Technol..

[19] Dargahi A., Sedaghati R., Rakheja S. (2019). On the properties of magnetorheological elastomers in shear mode: Design, fabrication and characterization. Compos. B. Eng..

[20] Na B.G., Chung K.H. (2018). Effect of precured EPDM on the property of magneto-rheological elastomer based on NR/EPDM blend. Elast. Compos..

[21] Kim T.W., Choi Y.J., Kim N.Y., Chung K.H. (2018). A study on the fatigue property of magneto-rheological elastomers. Elast. Compos..

[22] Zhou Y., Johnson M., Wen S., Betts A., Jerrams S. (2016). Equi-biaxial fatigue behaviour of magnetorheological elastomers in magnetic fields. J. Intell. Mater. Syst. Struct..

[23] Lian C., Lee K.H., Choi S.B., Lee C.H. (2018). A study of the magnetic fatigue properties of a magnetorheological elastomer. J. Intell. Mater. Syst. Struct..

[24] Wang Y., Gong X., Yang J., Xuan S. (2014). Improving the dynamic properties of MRE under cyclic loading by incorporating silicon carbide nanoparticles. Ind. Eng. Chem. Res..

[25] Gorman D., Murphy N., Ekins R., Jerrams S. (2016). The evaluation and implementation of magnetic fields for large strain uniaxial and biaxial cyclic testing of magnetorheological elastomers. Polym. Test..

[26] Johari M.A.F., Mazlan S.A., Nasef M.M., Ubaidillah U., Nordin N.A., Aziz S.A.A., Johari N., Nazmi N. (2021). Microstructural behavior of magnetorheological elastomer undergoing durability evaluation by stress relaxation. Sci. Rep..

[27] Yoon J.H., Lee S.W., Bae S.H., Kim N.I., Yun J.H., Ryu S.H. (2021). Effect of alignment of magnetic particles on the rheological properties of natural rubber composite. J. Polym. Res..

[28] Ubaidillah, Sutrisno J., Purwanto A., Mazlan S.A. (2015). Recent progress on magnetorheological solids: Materials, fabrication, testing, and applications. Adv. Eng. Mater..

[29] Bodelot L., Voropaieff J.P., Pössinger T. (2018). Experimental investigation of the coupled magneto-mechanical response in magnetorheological elastomers. Exp. Mech..

[30] Syam T., Muthalif A., Salem A., Hejazi A. (2020). 3D numerical modelling and analysis of a magnetorheological elastomer (MRE). J. Vibroengineering.

[31] Cantournet S., Desmorat R., Besson J. (2009). Mullins effect and cyclic stress softening of filled elastomers by internal sliding and friction thermodynamics model. Int. J. Solids Struct..

[32] Harwood J.A.C., Payne A.R. (1966). Stress softening in natural rubber vulcanizates. Part III. Carbon black-filled vulcanizates. J. Appl. Polym. Sci..

[33] Harwood J.A.C., Payne A.R. (1968). Hysteresis and strength of rubbers. J. Appl. Polym. Sci..

[34] Mullins L., Tobin N.R. (1957). Theoretical model for the elastic behavior of filler-reinforced vulcanized rubbers. Rubber Chem. Technol..

[35] Mullins L., Tobin N.R. (1965). Stress softening in rubber vulcanizates. Part I. Use of a strain amplification factor to describe the elastic behavior of filler-reinforced vulcanized rubber. J. Appl. Polym. Sci..

[36] Qi H.J., Boyce M.C. (2004). Constitutive model for stretch-induced softening of the stress–stretch behavior of elastomeric materials. J. Mech. Phys. Solids.

[37] Saintier N., Cailletaud G., Piques R. (2011). Cyclic loadings and crystallization of natural rubber: An explanation of fatigue crack propagation reinforcement under a positive loading ratio. Mater. Sci. Eng. A.

[38] Yu M., Fu J., Ju B.X., Zheng X., Choi S.B. (2013). Influence of x-ray radiation on the properties of magnetorheological elastomers. Smart. Mater. Struct..

[39] Fan Y., Gong X., Xuan S., Qin L., Li X. (2013). Effect of cross-link density of the matrix on the damping properties of magnetorheological elastomers. Ind. Eng. Chem. Res..

[40] Yang J., Gong X., Deng H., Qin L., Xuan S. (2012). Investigation on the mechanism of damping behavior of magnetorheological elastomers. Smart. Mater. Struct..

[41] Movahedi-Rad A.V., Keller T., Vassilopoulos A.P. (2019). Modeling of fatigue behavior based on interaction between time- and cyclic-dependent mechanical properties. Compos. Part A Appl. Sci. Manuf..

[42] Movahedi-Rad A., Keller T., Vassilopoulos A. (2018). Interrupted tension-tension fatigue behavior of angle-ply GFRP composite laminates. Int. J. Fatigue.

[43] Burhannuddin N.L., Nordin N.A., Mazlan S.A., Aziz S.A.A., Kuwano N., Jamari S.K.M., Ubaidillah (2021). Physicochemical characterization and rheological properties of magnetic elastomers containing different shapes of corroded carbonyl iron particles. Sci. Rep..

[44] Esmaeilnezhad E., Choi H.J., Schaffie M., Gholizadeh M., Ranjbar M. (2018). Polymer coated magnetite-based magnetorheological fluid and its potential clean procedure applications to oil production. J. Clean. Prod..

[45] Wang Y., Zhang X., Chung K., Liu C., Choi S.B., Choi H.J. (2016). Formation of core–shell structured complex microparticles during fabrication of magnetorheological elastomers and their magnetorheological behavior. Smart Mater. Struct..

[46] Dong X., Ma N., Qi M., Li J., Chen R., Ou J. (2012). The pressure-dependent MR effect of magnetorheological elastomers. Smart Mater. Struct..

[47] Jiang W., Yao J., Gong X., Chen L. (2008). Enhancement in magnetorheological effect of magnetorheological elastomers by surface modification of iron particles. Chinese J. Chem. Phys..

